# Ndr2 Kinase Controls Neurite Outgrowth and Dendritic Branching Through α_1_ Integrin Expression

**DOI:** 10.3389/fnmol.2018.00066

**Published:** 2018-03-06

**Authors:** Yunus E. Demiray, Kati Rehberg, Stefanie Kliche, Oliver Stork

**Affiliations:** ^1^Department of Genetics and Molecular Neurobiology, Institute of Biology, Otto-von-Guericke University Magdeburg, Magdeburg, Germany; ^2^Institute of Molecular and Clinical Immunology, Health Campus Immunology, Infectiology and Inflammation, Otto-von-Guericke University Magdeburg, Magdeburg, Germany; ^3^Center for Behavioral Brain Science, Magdeburg, Germany

**Keywords:** Ndr kinase, integrin inside-out signaling, α_1_ integrin, neurite outgrowth, dendritic branching, pheochromocytoma, primary hippocampal neurons

## Abstract

The serine/threonine kinase Ndr2 has been shown to control the inside-out activation of the β_1_subunit of integrins and the formation of neurites in both primary neurons and neurally differentiated pheochromacytoma (PC12) cells. In this study, we demonstrate that Ndr2 kinase furthermore determines the substrate specificity of neurite extension in PC12 cells via expression of α_1_β_1_ integrins. We show that stable overexpression of Ndr2 in PC12 cells increases neurite growth and extension on poly-D-lysine substrate, likely involving an increased expression of active β_1_ integrin in the growth tips of these cells. By contrast, the Ndr2 overexpressing cells do not show the α_1_β_1_ integrin-mediated enhancement of neurite growth on collagen IV substrate that can be seen in control cells. Moreover, they entirely fail to increase in response to activation of α_1_β_1_ integrins via a soluble KTS ligand and show a diminished accumulation of α_1_ integrin in neurite tips, although the expression of this subunit is induced during differentiation to comparable levels as in control cells. Finally, we demonstrate that Ndr2 overexpression similarly inhibits the α_1_β_1_ integrin-dependent dendritic growth of primary hippocampal neurons on laminin 111 substrate. By contrast, lack of Ndr2 impairs the dendritic growth regardless of the substrate. Together, these results suggest that Ndr2 regulates α_1_ integrin trafficking in addition to β_1_ integrin subunit activation and thereby controls the neurite growth on different extracellular matrix (ECM) substrates.

## Introduction

*Ndr2* (also termed serine/threonine kinase 38-like protein, STK38l) together with its close homolog *Ndr1*, as well as *Lats1/2* forms the conserved family of nuclear Dbf2-related (Ndr) kinases. As targets of the Hippo signaling pathway, these kinases are involved in gene regulation and regulation of cytoskeleton dynamics during cell division and differentiation (Emoto, 2011). Earlier studies showed that the Ndr kinase homolog in yeasts, *Dbf2p*, is essential for the polarization of cells during mitosis (Frenz et al., 2000). Further investigations in higher organisms demonstrated a prominent expression of Ndr2 and its homologs in neuronal tissues and a role in neuronal and brain development. While the Ndr2 homolog in *C. elegans*, *SAX-1*, is required for neuronal shape and neurite initiation, *Tricornered (Trc)* in *D. melanogaster* is important for dendritic branching/tiling (Zallen et al., 2000; Emoto et al., 2006). Our own work revealed prominent expression of Ndr2 in the central nervous system and an association with actin cytoskeleton in soma, neurites and spines in isolated hippocampal neurons (Rehberg et al., 2014). We could furthermore demonstrate an enhancement of neurite outgrowth through Ndr2 overexpression, using rat pheochromocytoma (PC12) cells as a model system (Stork et al., 2004). Further research confirmed that Ndr2 is indeed critical for neuronal polarization (Yang et al., 2014) and dendritic differentiation in mammalian neurons. Accordingly, Ndr2-deficient mice and rats show arbor specific alterations and premature branching in the hippocampus and neocortex (Ultanir et al., 2012; Rehberg et al., 2014). Addressing the cellular mechanisms of these functions, we could previously show that Ndr2 controls dendritic and axonal growth in mouse hippocampal neurons by affecting the activation state and endosomal trafficking of β_1_ integrin subunits to the dendritic surface (Rehberg et al., 2014).

Integrins are heterodimeric membrane receptors, consisting of one alpha and one beta subunit. Eighteen α and eight β subunits form 24 different integrin dimers with specific affinities to different extracellular ligands. These distinct integrin dimers have particular roles in regulating the attachment and spreading of the cells on various extracellular matrix (ECM) components (Stukel and Willits, 2016). In addition to mediating cell adhesion, integrins transmit information from the ECM and diffusible external growth cues in order to modulate the actin cytoskeleton and intracellular signaling pathways (Schmid and Anton, 2003; Calderwood, 2004). Their availability on the cell surface is an important control mechanism of integrin activity and integrin trafficking to the membrane is tightly regulated (Caswell et al., 2009; Bridgewater et al., 2012). Moreover, depending on both intra- and extracellular factors surface integrins can assume different conformational states with low, medium and high binding affinity (Humphries et al., 2003; Shattil et al., 2010). Neurons express a variety of integrin heterodimers which allow them to detect different ECM proteins; for example α_1_β_1_ and α_2_β_1_ bind to collagens, whereas α_6_β_1_ binds to laminin (Plow et al., 2000; Hynes, 2002). However, which intracellular mechanisms control the expression and distribution of integrin subtypes during neuronal development has remained largely unresolved so far.

Based on our previous findings implicating Ndr2 in the control of β_1_ integrin-dependent neurite growth, we here addressed the question whether this kinase modulates neurite outgrowth in an ECM-specific manner and examined the contribution of the α_1_ integrin subunit to this function. To this end we first used PC12 cells, which recruit different integrin subtypes to regulate their adhesion and neurite extension in response to ECM (Tomaselli et al., 1988; Lein et al., 2000) and provide a widely used model to examine to intracellular signaling related to neurite outgrowth (Harrill and Mundy, 2011). With a previously established line of Ndr2 overexpressing PC12 cells (Stork et al., 2004), we determined the differential effect of Ndr2 kinase on neurite growth on different ECM substrates. We further investigated how enhancement of integrin adhesion by divalent cations or a synthetic α_1_β_1_-specific KTS ligand affects the Ndr2-dependent neurite extension. Moreover, we determined the effects of Ndr2 overexpression on the amount of integrin subunits in the neurite tips of differentiating PC12 cells. Finally, we confirmed the role of Ndr2 in the substrate-specific regulation of primary hippocampal neurons dendritic growth. Our data provide evidence that Ndr2 kinase regulates both α_1_ integrin trafficking and β_1_ integrin activation to control the neurite morphology of the cell on different ECM substrates in PC12 cells and primary neurons.

## Materials and Methods

### Mice

Primary hippocampal neurons were derived from C57BL/6JBomTac mice (Taconic) mice, bred and raised in the animal facility at the Institute of Biology, Otto-von-Guericke University Magdeburg. Animal maintenance were done according to the guidelines of State of Saxony-Anhalt, Germany and approved by the Landesverwaltungsamt Sachsen-Anhalt (permission number: 42502-2-1177 Uni MD).

### Cell Culture

Rat pheochromocytoma (PC12) cells were maintained in RPMI medium supplemented with 10% horse serum, 5% fetal bovine serum and 100 U/mL Penicillin-Streptomycin (Gibco). EGFP expressing control PC12 cells (EGFP PC12) and EGFP-Ndr2 expressing PC12 cells (Ndr2 PC12) were previously established (Stork et al., 2004), stably transfected cells were maintained with 200 μg/ml G418 (Invitrogen) at 37°C under 5% CO_2_ incubation. For differentiation, cells were seeded (35,000 cells/cm^2^ for neurite growth assays and 5000 cells/cm^2^ for immunocytochemistry) on substrate coated glass coverslips (either with poly-d-lysine only (10 μg/cm^2^ in 0.15 M Borate buffer (pH 8.4), Sigma-Aldrich) or following substrates were added on poly-D-lysine (PDL) coated coverslips: fibronectin (3 μg/cm^2^ in water, Biochrom), laminin (5 μg/cm^2^ in serum-free RPMI media, BD Bioscience), gelatin (100 μg/cm^2^ in water, Biochrom), collagen I (10 μg/cm^2^ in 30% Ethanol, Sigma-Aldrich) and collagen IV (10 μg/cm^2^ in 30% Ethanol, Sigma-Aldrich) and cultured in low RPMI medium (RPMI with 0.2% horse serum, 1× PS and 200 μg/ml G418) supplemented with 50 ng/ml neurite growth factor (NGF, Gibco). To enhance general integrin binding, 0.3 mM MgCl_2_ were added to the low RPMI medium. In another set of experiments, a synthetic peptide bearing the α_1_β_1_ integrin-specific binding motif KTS (CW**KTS**LTSHYC, generated and purified by Pineda Antibody Service, Berlin, Germany) was applied to the cell media at a concentration of 140 μM to stimulate α_1_β_1_ integrin heterodimers specifically without altering the cell adhesion on collagen IV substrate (Moreno-Murciano et al., 2003).

### Neurite Growth Assays

After 4 days of NGF treatment, cells were briefly washed with phosphate buffered saline (PBS) and fixed with 4% para-formaldehyde (PFA)/Sucrose in PBS. Images of PC12 cells were taken using an inverted Axiovert 200 M microscope (Zeiss) at 10× objective under phase-contrast filter. Cells with neurites and cells with neurites longer than 100 μm were counted separately using AxioVision 4.0 (Zeiss) and values of each trial were normalized against PDL-control conditions.

### Immunocytochemistry

To stain for β_1_ integrin phosphorylated at threonine788/789 (β_1_^pThr788/789^), PC12 cells were stimulated with NGF as in neurite growth assays. Cells were fixed with 4% PFA/sucrose in PBS and permeabilized with 0.3% TritonX for 10 min at room temperature. Coverslips were incubated with 5% bovine serum albumin (Roth) and 5% donkey serum (Linaris) for 2 h at room temperature to block unspecific binding. Immunocytochemistry was done using a pThr^788/789^ β_1_ integrin antibody (1:200, Abcam ab5189). Cells were incubated with the primary antibody overnight at 4°C, washed with PBS and incubated with Alexa-conjugated secondary antibodies (1:1000 dilution in PBS, Invitrogen) for 1 h at room temperature. For staining of α_1_ integrin, PC12 cells were grown for a prolonged period of 6 days, according to Zhang et al. (1993), to allow for high expression levels. Cells were fixed, permeabilized and stained using α_1_ integrin primary antibody (Abcam, ab78479; diluted 1:200 in PBS). Alexa-coupled secondary antibody was applied together with rhodamine-phalloidine (1:40, R415- Molecular Probes) according to the manufacturer’s protocol to detect the actin cytoskeleton. Cells were finally mounted to slide glasses using ImmuMount (Thermo Scientific) and images were captured with Meta 510 (Zeiss) and DMI 6000 (Leica) microscopes. Images were randomized and evaluated by a researcher who was blinded with respect to the cell line. The most prominent growth tip of differentiated PC12 cells was selected and fluorescent intensities in the growth tips and cell bodies were quantified using ImageJ (NIH) software with built-in histogram and measure functions.

### Western Blotting

NGF-treated PC12 cells (0 day, 3 days and 6 days NGF treatment (50 ng/ml)) were briefly washed with pre-warmed PBS and lysed in lysis buffer (1% lauryl maltoside N-dodycyl-β-D-maltoside (Merck), 1% NP-40 (Sigma Aldrich), 1 mM Na-orthovanadate, 2 mM EDTA, 50 mM Tris-HCl, 150 mM NaCl, 0.5% deoxycholic acid, 1 mM 4-(2-Aminoethyl)benzenesulfonyl fluoride hydrochloride, 1 μM Pepstatin A, 1 mM NaF and protease inhibitor cocktail (one tablet per 50 ml, Pierce)). Proteins were separated according to their size on polyacrylamide gels and transferred to PVDF membranes (Immobilon FL, Millipore). Membranes were blocked with 5% milk powder (Roth) in PBS for 2 h in room temperature and incubated with α_1_ integrin (1:1000 dilution in PBS+0.1% Tween; Abcam78479) and α-tubulin (1:5000 dilution in PBS+0.1% Tween; Sigma Aldrich T6199) antibodies overnight at 4°C. Membranes were briefly washed with PBS and fluorescently labeled IRDye secondary antibodies (Licor) was used with 1:15,000 dilution in PBS+0.1% Tween. After 1 h incubation at room temperature, membranes were washed three times with PBS+0.2% Tween and visualized using an Odyssey scanner (Licor).

### Primary Cell Culture

Hippocampi from embryonic day 18 (E18) to E19 mice were dissected and disassociated using MACS Neural Tissue Dissociation Kit (Milteny). Disassociated cells were plated either on poly-D-lysine (10 μg/cm^2^, Sigma-Aldrich) or poly-D-lysine+ Laminin111 (0.45 μg/cm^2^, in PBS, Biolamina) coated coverslips (25,000 cells/cm^2^). Except for the plating media (DMEM+ 10% FBS (v/v) + L-GlutaMax (2 mM)) at day *in vitro* (DIV0), neurons were kept with neurobasal media containing B27 (2% v/v) and L-GlutaMax (0.5 mM) (all from Invitrogen) that had been glia conditioned for 72 h. At DIV3, neurons were transfected either with EGFP-C1 (ClonTech), EGFP-Ndr2 (Stork et al., 2004), pLL3.7_shLuc (TTG GAG AAA AGC CTT GTT T) or pLL3.7_shNdr2 (CCT CAT CTG CCA ATC CCT C; Rehberg et al., 2014) using CalPhos Mammalian Transfection kit according to the manufacturer instructions (ClonTech). All neurons were also co-transfected with pCMV-tdTomato (ClonTech) to outline the dendritic morphology. At DIV7, primary neurons were fixed with 4% paraformaldehyde and 4% sucrose in 0.1 M PBS, mounted on slides using Immuno-Mount (Thermo Scientific) and images were taken using a DMI 6000 epifluorescence microscope (Leica). Dendrites of the double-transfected neurons were selected according to their morphological features (Kaech and Banker, 2006) and were traced using QWin software (Leica). Sholl analysis of the traced dendrites was done using Fiji (ImageJ) with 10 μm radius intervals.

### Statistics

Statistical analysis was performed using two-way ANOVA followed by Fisher Least Significant (LSD) test or Kruskal-Wallis test for multiple comparisons. Immunofluorescence data were log-transformed before statistical analysis. The number of cells with long neurites (>100 μm) and short neurites (<100 μm) were compared using Chi-square tests. Pairwise comparisons were done using Student’s *t*-test or Mann-Whitney test when appropriate and a statistical threshold for significance was set at *p* < 0.05. Normality of the datasets was tested using Shapiro-Wilk test.

## Results

### Ndr2 Is Involved in PC12 Cells Neurite Extension on Different Substrates

**Figure 1:**
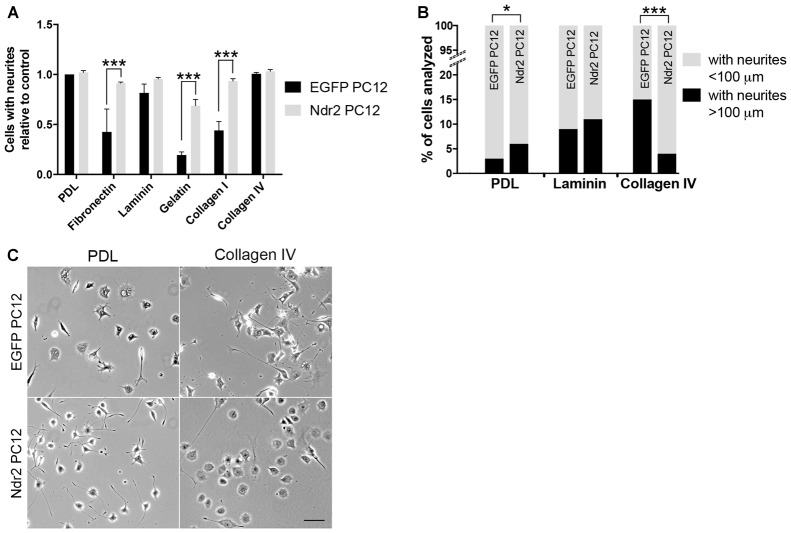
Substrate-specific modulation of neurite outgrowth through Nuclear Dbf2-related 2 (Ndr2). **(A)** The proportion of cells with neurites is increased on fibronectin, gelatin and collagen I in Ndr2 PC12 cells, while the more permissive substrates poly-D-lysine (PDL), laminin and collagen IV show generally high neurite formation in both Ndr2 rat pheochromocytoma cells (PC12) and EGFP PC12 control cells (mean + SEM, *N* = 3, two-way ANOVA genotype × substrate interaction: *F*_(5,24)_ = 4.575, *p* = 0.0045). **(B)** In line with a generally increased neurite growth, the Ndr2 PC12 cells also show an increase in the proportion of cells bearing long neurites (>100 μm) on PDL substrate (*X*^2^_(1)_ = 4.276, *p* < 0.05). However, the Ndr2 PC12 cells fail to show the enhancement of neurite extension on collagen IV substrate that is evident in EGFP PC12 controls (*X*^2^_(1)_ = 81.45, *p* < 0.001). **(C)** Microscopic images of EGFP PC12 and Ndr2 PC12 cells on PDL and Collagen IV substrates displaying the differences in neurite numbers and morphologies. *p < 0.05; ***p < 0.001. Scale bars: 100 μm.

To investigate the role of Ndr2 kinase in neurite growth, EGFP transfected PC12 cells (EGFP PC12) and Ndr2 transfected PC12 cells (Ndr2 PC12) were cultured on different ECM substrates and treated with NGF. Both cell lines showed efficient neurite formation on PDL and collagen IV with >95% of cells forming discernable neurites under each condition. Laminin also was a favorable substrate for both lines, whereas less than 50% of control cells showed neurites on fibronectin, collagen I and gelatin. On those less efficient substrates, an increase of outgrowth in Ndr2 cells was evident, as the proportion of cells with neurites were significantly increased on fibronectin, gelatin and collagen I substrates (Figure 1A, two-way ANOVA genotype × substrate interaction: *F*_(5,24)_ = 4.575, *p* = 0.0045). To address potential differences in neurite extension, we further analyzed cells with neurites longer than 100 μm (Figure 1B). Here we have selected PDL, Laminin and Collagen IV substrates since they resulted in neuronal differentiation in more than 90% of cells in both cell lines. Here we confirmed the previously reported increase in Ndr2 PC12 cells neurite extension of on PDL (*X*^2^_(1)_ = 4.276, *p* < 0.05, Stork et al., 2004), but did not observe any change in neurite extension on laminin substrate between genotypes (*X*^2^_(1)_ = 0.1364, *p* = 0.71). In contrast to PDL, on collagen IV substrate Ndr2 PC12 cells displayed significantly reduced rather than increased neurite growth compared to EGFP PC12 cells (Figures 1B,C; *X*^2^_(1)_ = 81.45, *p* < 0.001). Therefore, PDL and collagen IV were chosen as substrates for further experiments to investigate how Ndr2 kinase controls substrate specific neural differentiation of PC12 cells.

### Ndr2 Controls the β_1_ Integrin Activity in Neurite Tips

**Figure 2:**
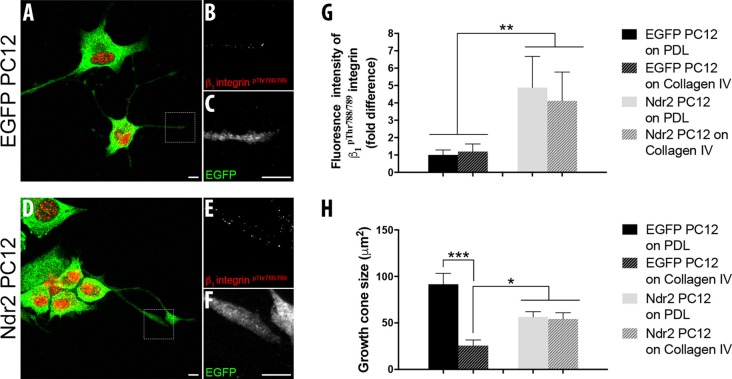
Ndr2 increases the expression of activated β_1_ integrins at neurite tips regardless of the extracellular matrix (ECM) substrate. Immunocytochemistry of differentiating EGFP PC12 cells **(A–C)** and Ndr2 PC12 cells **(D–F)**. A closer look into the growth cones of Ndr2 PC12 cells reveals the co-localization of activated β_1_ integrin (pThr^788/789^) **(E)** and Ndr2 **(F)** at neurite tips. Scale bars: 10 μm. **(G)** Quantitative analysis of fluorescence intensity (*n* = 10 cells per group) reveals a significant accumulation of activated β_1_ integrin (pThr^788/789^) in neurite tips of differentiating Ndr2 PC12 cells compared to EGFP PC12 cells, both on PDL and collagen IV (two-way ANOVA genotype effect: *F*_(1,36)_= 7.407, *p* < 0.01). **(H)** Decreased growth cone size of control cells, but not of Ndr2 PC12 cells on collagen IV substrate (two-way ANOVA genotype × substrate interaction: *F*_(1,35)_ = 15.67, *p* = 0.0004). Values are mean + SEM; *p < 0.05; **p < 0.01; ***p < 0.001.

Integrin receptors on the cell surface are recruited by these ECM substrates and previous studies showed that β_1_ integrin containing integrin heterodimers are the main receptors for their signal transmission in developing neurons (Denda and Reichardt, 2007; Lei et al., 2012). Since we have previously described the role of Ndr2 in the “inside-out” activation of β_1_ integrins, we next tested whether this process may be involved in the differential response of Ndr2 PC12 cells to integrin substrates. Immunohistochemical staining of β_1_^pThr788/789^, which is required for β_1_ integrin activation (Nilsson et al., 2006), revealed an expression of this activated β_1_ integrin form in both somata and neurite tips of both control cells and Ndr2 PC12 cells (Figures 2A–F). Quantification of the relative amount of β_1_^pThr788/789^ revealed no main effect of the coating substrate (two-way ANOVA, substrate effect: *F*_(1,36)_ = 0.05323, *p* = 0.8188). However, an increase of β_1_^pThr788/789^ was observed in the neurite tips of Ndr2 PC12 cells compared to control cells (two-way ANOVA genotype effect: *F*_(1,36)_= 7.407 *p* < 0.01), regardless of the coating substrate (genotype × substrate interaction: *F*_(1,36)_= 0.1485, *p* = 0.7022; Figure 2G). Furthermore, while collagen IV substrate decreased the growth cone size of control cells, Ndr2 PC12 cells did not change their growth cone size upon collagen IV coating (genotype × substrate interaction: *F*_(1,35)_ = 15.67, *p* = 0.0004; Figure 2H). However, β_1_^pThr788/789^ raw fluorescence results were independent of the growth cone sizes, since fluorescence intensity of β_1_^pThr788/789^ per μm^2^ of growth cones revealed only a genotype difference between EGFP and Ndr2 PC12 cells as in raw fluorescence results (two-way ANOVA genotype effect: *F*_(1,36)_= 4.867, *p* = 0.0338; data not shown).

### Ndr2 Impairs α_1_β_1_ Integrin-Mediated Neurite Extension

**Figure 3:**
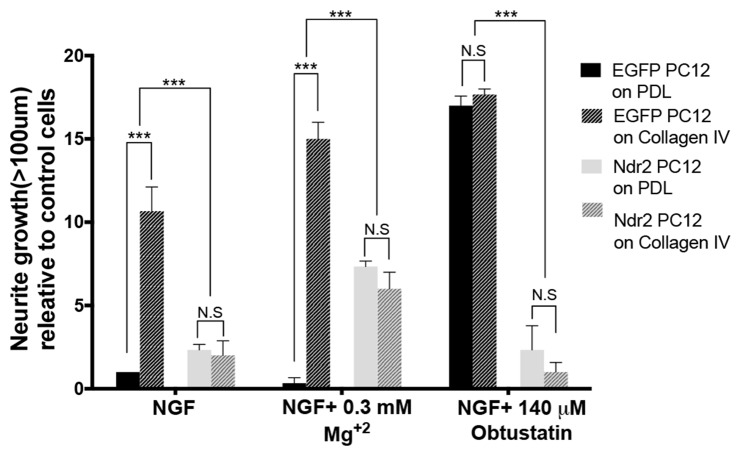
Lack of α_1_β_1_ integrin activation to stimulate outgrowth in Ndr2 overexpressing cells. Without additional integrin stimulation, control cells but not Ndr2 PC12 cells show an enhanced growth on collagen IV compared to PDL. Unspecific enhancement of integrin adhesion by 0.3 mM Mg^2+^ somewhat increases the neurite growth of Ndr2 PC12 cells on both PDL and Collagen IV, however, without reaching the high levels seen on collagen IV with control cells. The synthetic KTS ligand obtustatin induces growth in control cells on PDL to the same high level as on collagen IV. However, it does not stimulate growth of Ndr2 PC12 cells on either substrate. In summary, α_1_β_1_ stimulation by collagen IV substrate or a soluble KTS ligand stimulates neurite growth only in control cells, but not in Ndr2 PC12 (two-way ANOVA genotype × treatment interaction: *F*_(6,24)_= 41.05 *p* < 0.001). Values are mean + SEM; *N* = 3; ***p < 0.001; N.S *p* > 0.05.

α_1_β_1_ integrin heterodimers are the main receptors on the cell surface that respond to collagen IV substrate and α_1_β_1_ integrins show increased binding to collagen IV, rather than collagen I, during neurite extension of human neuroblastoma cells (Carmeliet et al., 1994). To identify the recognition process that underlies the growth deficiency on collagen IV, we tested the effect of integrin stimulation via Mg^2+^ and a KTS ligand specific to α_1_β_1_ integrins on PDL or Collagen IV substrates. Here we observed a general enhancement of outgrowth in the Ndr2 PC12 cells by Mg^2+^, but a complete failure to increase growth by the α_1_β_1_ integrin ligand. A significant interaction was found between Ndr2 expression and integrin stimulation (Figure 3, genotype × treatment interaction: *F*_(6,24)_= 41.05 *p* < 0.001).

As in the previous experiment a significant enhancement of neurite growth by collagen IV was observed in EGFP PC12 control cells, but not by Ndr2 PC12 cells upon mere NGF stimulation. Under 0.3 mM Mg^2+^ treatment along with NGF, EGFP PC12 cells slightly further increased growth on collagen IV, while Ndr2 cells increased growth significantly regardless of the substrate, but remained lower than control cells on collagen IV. To specifically address α_1_β_1_ integrin function we synthesized a KTS ligand peptide (CW**KTS**LTSHYC), derived from the larger disintegrin obtustatin (Marcinkiewicz et al., 2003; Moreno-Murciano et al., 2003) and applied it to the cell culture medium. At the concentration used in our experiments (140 μM; based on Marcinkiewicz et al., 2003), this KTS ligand did not induce any change in the flattening of the cells that would indicate a generally altered adhesion (Kruskal-Wallis test between treatments: *p* = 0.93), but a maximal neurite growth of PC12 control cells on both PDL and collagen IV was observed. As a side note, under 140 μM obtustatin treatment, no significant difference was observed between control cells plated on PDL and Collagen IV, suggesting that obtustatin peptide did not cause any inhibition on Collagen IV substrate. In Ndr2 PC12 cells, however, in spite of the increased expression of β_1_ integrin phosphorylation (Thr^788/799^) in the neurite tips (Figure 2G), the KTS agonist peptide like collagen IV entirely failed to induce neurite growth (Figure 3).

### Ndr2 Modulates α_1_ Integrin Distribution During Neurite Growth

**Figure 4:**
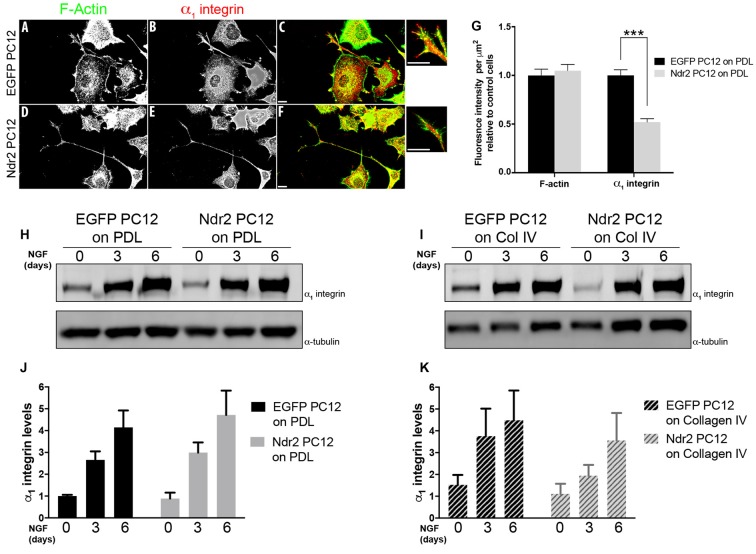
Reduced localization of α_1_ integrin to neurite tips. **(A,B)** Microscopic images of 6 day neurite growth factor (NGF) treated PC12 cells showing the expression and distribution F actin and of the α_1_ integrin receptor in control and **(D,E)** Ndr2 PC12 cells. **(C,F)** Overlay image. Scale bars: 10 μm. **(G)** Quantification of labeling at neurite tips (*n* = 46 for EGFP PC12, 49 for Ndr2 PC12 cells) reveals a significant reduction of α_1_ integrin levels in Ndr2 PC12 cells compared to EGFP PC12 cells (two-tailed Student’s *t*-test, *p* < 0.001), but no change in F-actin (two-tailed Student’s *t*-test, *p* = 0.595). **(H,I)** Immunoblotting shows the elevated expression of α_1_ integrin in both cell lines upon 3 days or 6 days of NGF treatment on both substrates. **(J,K)** Quantification of the blots shows that α_1_ integrin protein levels increases similarly during differentiation in EGFP PC12 and Ndr2 PC12 cells (*n* = 4 each; on PDL: two-way ANOVA, genotype effect: *F*_(1,18)_= 0.2754, *p* = 0.6062; on Collagen IV: two-way ANOVA, genotype effect: *F*_(1,18)_= 1.74, *p* = 0.2036). Values are mean + SEM; ***p < 0.001.

As those data indicated a specific impairment of α_1_β_1_ integrin function in Ndr2 PC12 cells, next we examined the expression and distribution of the α_1_ integrin subunit during differentiation (Figures 4A–F). In fact, we observed that Ndr2 PC12 cells show significantly less α_1_ integrin labeling at their neurite tips than EGFP PC12 cells (Figure 4G, two-tailed Student’s *t*-test, *p* < 0.001). At the same time, labeling for F-actin was not different in the neurite tips. To distinguish whether Ndr2 regulates the α_1_ integrin trafficking or its overall expression, we also determined the total α_1_ integrin expression in EGFP PC12 and Ndr2 PC12 cells (Figures 4H,I). Following either 3 days or 6 days of NGF treatment, a profound increase in α_1_ integrin levels was observed that was as pronounced in Ndr2 PC12 cells as in EGFP PC12 controls, similarly on PDL (two-way ANOVA, genotype effect: *F*_(1,18)_= 0.2754, *p* = 0.6062) and Collagen IV (two-way ANOVA, genotype effect: *F*_(1,18)_= 1.74, *p* = 0.2036) substrates (Figures 4J,K).

### Ndr2 Controls Dendritic Branching in Primary Neurons

**Figure 5:**
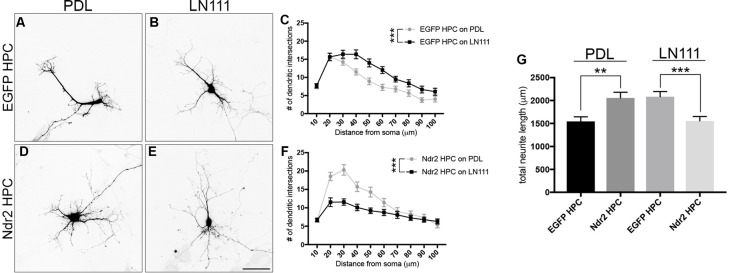
Overexpression of Ndr2 kinase impairs α_1_β_1_-dependent dendritic growth of hippocampal primary neurons. Primary hippocampal neurons transfected with EGFP plasmid and plated on **(A)** PDL and **(B)** Laminin-111 (LN111). **(C)** Sholl analysis of their dendrites reveals that LN111 substrate increases the dendrite branching significantly compared to PDL coating (two-way ANOVA between substrates: *F*_(1,57)_= 12.59, *p* = 0.0008, *n* = 30 each). **(D–F)** On the other hand, when Ndr2 overexpressing neurons are plated on the same substrates, Sholl analysis shows higher dendritic branching of Ndr2 hippocampal primary culture (HPC) on PDL rather than LN111 substrate (two-way ANOVA between substrates: *F*_(1,57)_= 15.46, *p* = 0.0002, *n* = 29 for PDL and 30 for LN111). **(G)** Total dendritic length of Ndr2 HPC is increased compared to EGFP PC12 cells on PDL and but reduced on LN111 substrate (one-way ANOVA: *F*_(3,115)_ = 7.72, *p* < 0.0001). Values are mean + SEM; **p < 0.01; ***p < 0.001.

To investigate the role of Ndr2 in neuronal differentiation, we acutely transfected primary hippocampal neurons with Ndr2 constructs (overexpression and silencing) on different substrates. Neurons were plated on coverslips that are coated either with PDL or Laminin-111 (LN111), a previously established ECM molecule of primary neurons and a known α_1_β_1_ integrin substrate (Desban et al., 2006; Tulla et al., 2008). When control neurons (EGFP hippocampal primary culture (HPC)) were plated on LN111, their dendritic branching significantly increased compared to PDL (two-way ANOVA substrate effect: *F*_(1,57)_= 12.59, *p* = 0.0008; Figures 5A–C). However, Ndr2 overexpressing neurons (Ndr2 HPC) showed less dendritic branching on LN111 compared to PDL (two-way ANOVA substrate effect: *F*_(1,57)_= 15.46, *p* = 0.0002; Figures 5D–F), suggesting an impairment of α_1_ integrin dependent growth, in line with our PC12 cell results. Furthermore, analysis of the total dendritic length of these acutely-transfected neurons revealed that Ndr2 overexpression increases total dendritic length on PDL, while it reduces the total dendritic length on LN111 compared to the control neurons (one-way ANOVA: *F*_(3,115)_ = 7.72, *p* < 0.0001; Figure 5G).

**Figure 6:**
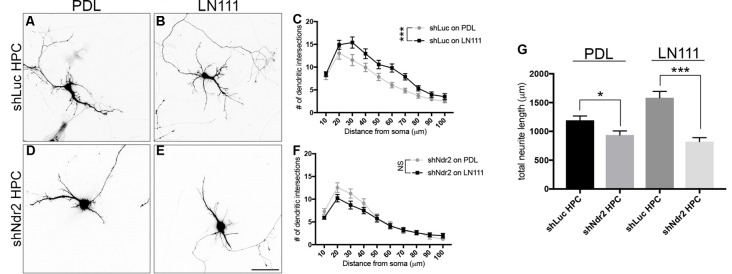
Ndr2 kinase is required for LN111 enhancement of dendritic growth in hippocampal neurons. Primary hippocampal neurons transfected with shLuc plasmid and plated on **(A)** PDL and **(B)** LN111. **(C)** Sholl analysis of their dendrites reveals that LN111 substrates increase the dendrite branching significantly compared to PDL coating (two-way ANOVA between substrates: *F*_(1,54)_= 12.16, *p* = 0.0010, *n* = 26 for PDL and 30 for LN111). **(D–F)** On the other hand, when Ndr2 kinase is silenced, dendritic morphology of the neurons are unchanged between substrates (two-way ANOVA between substrates: *F*_(1,58)_ = 1.262, *p* = 0.2660, *n* = 30 for PDL and 31 for LN111). **(G)** In fact, silencing of Ndr2 kinase expression results in shorter total dendrite length compared to control neurons on both PDL and LN111 substrates (one-way ANOVA: *F*_(3,113)_= 17.33, *p* < 0.0001). Values are mean + SEM; *p < 0.05; ***p < 0.001.

Finally, we tested how silencing of Ndr2 kinase in neurons affects the morphology of dendrites. shLuc transfected neurons (shLuc HPC) serving as controls increased their dendritic branching upon LN111 substrate as expected (two-way ANOVA substrate effect: *F*_(1,54)_= 12.16, *p* = 0.0010; Figures 6A–C). By contrast, Ndr2 knockdown neurons (shNdr2 HPC) entirely failed to increase their growth in response to LN111 substrates (two-way ANOVA substrate effect: *F*_(1,58)_ = 1.262, *p* = 0.2660; Figures 6D–F). In fact, total dendritic lengths of shNdr2 neurons were significantly shorter than shLuc neurons on both PDL and LN111 substrates (one-way ANOVA: *F*_(3,113)_= 17.33, *p* < 0.0001; Figure 6G).

## Discussion

Neurite outgrowth is critically dependent on guidance signals such as the ECM being detected via integrins. Focal adhesion points are observed at the tip of filopodia evading the growth cone of primary neurons and on the neurite tips of differentiating PC12 cells (Eva et al., 2010). However, how cells control integrin levels and assemble specific α_1_β_1_ integrin heterodimers at these sites is still not completely understood. Our current data demonstrate that the serine/threonine kinase Ndr2 is involved in these processes in neurally differentiating PC12 cells. Ndr2 not only enhances the accumulation of β_1_ integrins in neurite tips of these cells but also reduces the incorporation of α_1_ subunits, resulting in an altered neurite outgrowth on different integrin substrates. α_1_ integrin subunits are only weakly expressed during the induction of neurite outgrowth, but increase at later stages during neurite extension and stabilization. Correspondingly, Ndr2 PC12 cells, although efficiently forming neurites on various substrates including collagen IV, showed a reduced collagen IV-induced growth of long neurites (>100 μm). This substrate-specific effect could also be observed in primary hippocampal neurons, where overexpression of Ndr2 prevented LN111 induced dendritic growth. Loss of Ndr2 by contrast generally inhibited dendritic growth in line with previous observations (Ultanir et al., 2012; Rehberg et al., 2014) thus also preventing the LN111 induced growth effect.

The ECM during neuron development supports the attachment of neurons and provides guidance cues for the growing neurites (Myers et al., 2011). Although it is a heterogenous mixture of different glycoproteins and proteoglycans, different ECM substrates can be classified into three major groups according to their integrin recognition sequences: laminins, fibronectins (RGD sequence containing) and collagens (GFOGER sequence containing; Barczyk et al., 2010). The basal lamina component collagen IV is furthermore characterized by its KTS sequence, which is recognized by α_1_β_1_ integrins (Kisiel et al., 2004). Laminin subtype 111 (also known as laminin-1) is another major component of basal lamina (Fiore et al., 2017) controls the migration and spreading of neural crest cells via α_1_β_1_ integrin receptors on the membrane (Desban et al., 2006). Earlier studies showed that each of the above-mentioned ligands or their combinations improve the survival/neurite growth of differentiating neurons (O’Connor et al., 2001; Fusaoka-Nishioka et al., 2011; Tonge et al., 2012).

A total of 28 different collagen subtypes have been described so far (Gordon and Hahn, 2010; Rozario and DeSimone, 2010) of which the collagen subtypes I, II, III and IV can induce neurite extension of PC12 cells in a Mg^2+^ dependent manner (Turner et al., 1987). Collagen I and IV were also shown to promote neuronal differentiation and neurite growth of rat cortical neurons (Ali et al., 1998; O’Connor et al., 2001). Since divalent ions prime integrin heterodimers to their activation states, the observed moderate enhancement of neurite growth in Ndr2 PC12 under the 0.3 mM Mg^2+^ conditions thus is in line with a mediation by integrin binding (Tiwari et al., 2011) and likely due to the increase of phosphorylated integrin (β_1_^pThr788/789^) in their neurite tips.

Thr^788/789^ site at the cytosolic domain of integrin β_1_ is required for its activation (Nilsson et al., 2006). Ndr2 has previously been shown to stimulate phosphorylation at Thr^788/789^ of β_1_ integrins via an intermediary kinase and to enhance their recycling to the cell membrane. In primary hippocampal neurons, Ndr2 thus mediates the “inside out” activation of dendritic integrins and stimulates dendritic growth and branching (Rehberg et al., 2014). Our current data further add to those findings, as we can show that silencing the Ndr2 kinase impairs the dendrite growth of hippocampal neurons regardless of the substrate (Figure 6G). Moreover, phosphorylation at the Thr^788/789^ activation site prevents internalized integrins from lysosomal degradation and favors recycling (Böttcher et al., 2012); accordingly, Ndr2 is associated with Rab5- and Rab11-positive early and late recycling endosomes in neurons and in PC12 cells (Rehberg et al., 2014). In the current study, we could demonstrate that this mechanism works in PC12 cells independently of the ECM substrate (PDL or collagen IV). In neuronal cells, β_1_subunit containing heterodimers are the major receptors for responding to those ECM substrates (Luo et al., 2007; Stukel and Willits, 2016). Previous studies also showed that outside-in stimulation of β_1_ integrin receptor increases the dendritic branching and its inhibition has the adverse effect on neuron morphology (Moresco et al., 2005; Marrs et al., 2006; Schlomann et al., 2009). This effect of β_1_ integrins on dendritic arborization is particularly evident in early development and differentiation (Warren et al., 2012).

We therefore considered that the disturbance of collagen IV- induced outgrowth in Ndr2 PC12 may have been related to an increased adhesion to the substrate and associated changes in “outside-in” signaling. We addressed this question and the specificity of the observed effects by applying a diffusible ligand mimicking collagen IV-mediated binding to integrins. While collagen IV substrate can be recognized by both α_1_β_1_and α_2_β_1_ integrins, previous studies showed that α_1_β_1_ integrin heterodimer has higher affinity and is the primary receptor that interacts with collagen IV (Kern et al., 1993; Knight et al., 2000; Becker et al., 2013). Furthermore, studies in T cells, which rely mainly on integrin receptors for adhesion, showed that α_1_β_1_ integrin expressing cells are enriched on collagen IV surfaces while α_2_β_1_expressing cells tends to localize on collagen I rich spaces (Richter et al., 2007). Strikingly, while the α_1_β_1_ integrin ligand obtustatin mimicked the collagen IV-induced neurite growth in control cells, Ndr2 overexpressing PC12 cells entirely failed to respond to the ligand.

This strongly suggests that the reduced collagen IV response of Ndr2 PC12 cells may be caused by their reduced expression of α_1_ integrin in neurite tips (Figures 4A–G). It has been reported that α_1_ integrin expression levels increase in PC12 cells upon NGF treatment, correlating with the adhesion on collagen and neurite extension levels of the cell (Zhang et al., 1993). As we detected similar induction of α_1_ integrin expression in Ndr2 PC12 cells and EGFP PC12 cells (Figures 4H,I), our findings indicate a disturbance of α_1_ integrin trafficking rather than expression regulation in Ndr2 PC12 cells.

It is plausible to assume that the Ndr2 kinase may regulate the α integrin trafficking via Rab-dependent transport mechanisms. We have previously demonstrated the association of Ndr2 with Rab5 and Rab11 positive integrin recycling vesicles (Rehberg et al., 2014) and the Rab-8 guanine exchange factor, Rabin8 has been identified as a substrate for Ndr kinases (Ultanir et al., 2012). Rabin8 stimulates endosomal transport in PC12 cells in Rab11-dependent manner and is required for neurite outgrowth in these cells (Homma and Fukuda, 2016). Previous research further demonstrated that α integrin subunits are internalized and recycled via Rab-mediated pathways and that Rab-association domains are conserved among α integrins (Pellinen et al., 2008; Caswell et al., 2009). A differential α subunit trafficking may also involve clathrin-adaptor proteins acting as intermediate regulators of vesicle trafficking (Bridgewater et al., 2012). For example, the endocytic adaptor protein Dab2 was shown to bind to and increase endocytosis of α_1_, α_2_ and α_3_ integrin subunits (Teckchandani et al., 2009). On the other hand α_2_ and α_6_, but not α_1_ integrin contain binding motifs for the adapter-protein 2 (AP-2, De Franceschi et al., 2016), which is redistributed to the membrane in PC12 cells after NGF treatment (Beattie et al., 2000). Intriguingly, Ndr2 kinase can phosphorylate and regulate the activity of AP-2 associated kinase (AAK1), which controls the AP-2 binding affinity and dendritic growth of rat hippocampal neurons (Ultanir et al., 2012).

Our data suggest, that in addition to enhancing β_1_ Thr^788/789^ phosphorylation and recovery, Ndr2 controls the selective trafficking of α_1_ subunit-containing integrins. While β subunits of integrins interact with intracellular proteins and are required for activation of downstream signaling cascades, α subunits critically determine the extracellular ligand specificity of the receptor (Barczyk et al., 2010; Stukel and Willits, 2016). Accordingly, β_1_ integrin expression is rather steady and uniform compared to α subunits which are both temporally and spatially regulated in developing cortex (Heino et al., 1989; Schmid and Anton, 2003). As integrin subunits need to form stable αβ heterodimers to exit the endoplasmic reticulum and to join the membrane trafficking/recycling pathways (Bouvard et al., 2013), α subunits appear to serve as limiting and specifying factors that determine the rate of dimerization with the β subunits as well as the integrin heterodimer sorting (Heino et al., 1989). Ndr2, by differentially regulating β_1_ integrin recycling and the surface localization of α_1_ integrin subunit localization, thus determines the substrate specificity of neurite outgrowth in PC12 cells and hippocampal neurons.

## Author Contributions

KR, SK and OS designed the research. KR and YED performed the experiments. YED and OS wrote the article.

## Conflict of Interest Statement

The authors declare that the research was conducted in the absence of any commercial or financial relationships that could be construed as a potential conflict of interest.

## Abbreviations

AP-2: Adapter-protein 2
EGFP PC12: EGFP expressing control PC12 cells
Ndr2 PC12: EGFP-Ndr2 expressing PC12 cells
ECM: Extracellular matrix
HPC: Hippocampal primary culture
LN111: Laminin-111
NGF: Neurite growth factor
Ndr: Nuclear Dbf2-related
PDL: poly-D-lysine
PC12: Rat pheochromocytoma cells.
